# Supplementary material for: Synthesis and Antimicrobial Activity of Short Analogues of the Marine Antimicrobial Peptide Turgencin A: Effects of SAR Optimizations, Cys-Cys Cyclization and Lipopeptide Modifications
Source: Int J Mol Sci. 2022 Nov 10;23(22):13844. doi: 10.3390/ijms232213844 (PMC9696794; doi:10.3390/ijms232213844)
Supplement: Supplementary file 1 [file ijms-23-13844-s001.zip › ijms-2009707-supplementary.pdf]

Supporting information

Synthesis and Antimicrobial Activity of Short Analogues of the Marine Antimicrobial Peptide Turgencin A: Effects of SAR Optimizations, Cys-Cys Cyclization And lipopeptide Modifications

Hymonti Dey [1,†], Danijela Simonovic [2,†], Ingrid Norberg-Schulz Hagen [2], Terje Vasskog [2], Elizabeth G. Aarag Fredheim [2], Hans-Matti Blencke [1], Trude Anderssen[2], Morten B. Strøm [2,*] and Tor Haug [1,*]

[1] The Norwegian College of Fishery Science, Faculty of Biosciences, Fisheries and Economics, UiT The Arctic University of Norway, NO-9037 Tromsø, Norway
[2] Department of Pharmacy, Faculty of Health Sciences, UiT The Arctic University of Norway, NO-9037 Tromsø, Norway
* Correspondence: morten.strom@uit.no (M.B.S.); tor.haug@uit.no (T.H.)
† These authors contributed equally to this work.

Contents:

Figure S1. UPLC chromatogram of purified cyclic peptide **cTurg-1**.
Figure S2. UPLC chromatogram of purified cyclic peptide **cTurg-2**.
Figure S3. UPLC chromatogram of purified cyclic peptide **cTurg-3**.
Figure S4. UPLC chromatogram of purified cyclic peptide **cTurg-4**.
Figure S5. UPLC chromatogram of purified cyclic peptide **cTurg-5**.
Figure S6. UPLC chromatogram of purified cyclic peptide **cTurg-6**.
Figure S7. UPLC chromatogram of purified cyclic peptide **cTurg-7**.
Figure S8. UPLC chromatogram of purified linear lipopeptide **C_8-Turg-1**.
Figure S9. UPLC chromatogram of purified linear lipopeptide **C_{10}-Turg-1**.
Figure S10. UPLC chromatogram of purified linear lipopeptide **C_{12}-Turg-1**.
Figure S11. UPLC chromatogram of purified linear lipopeptide **C_8-Turg-2**.
Figure S12. UPLC chromatogram of purified linear lipopeptide **C_{10}-Turg-2**.
Figure S13. UPLC chromatogram of purified linear lipopeptide **C_{12}-Turg-2**.
Figure S14. UPLC chromatogram of purified linear lipopeptide **C_8-Turg-6**.
Figure S15. UPLC chromatogram of purified linear lipopeptide **C_{10}-Turg-6**.
Figure S16. UPLC chromatogram of purified linear lipopeptide **C_{12}-Turg-6**.
Figure S17. UPLC chromatogram of purified cyclic lipopeptide **C_8-cTurg-1**.
Figure S18. UPLC chromatogram of purified cyclic lipopeptide **C_{10}-cTurg-1**.
Figure S19. UPLC chromatogram of purified cyclic lipopeptide **C_{12}-cTurg-1**.
Figure S20. UPLC chromatogram of purified cyclic lipopeptide **C_8-cTurg-2**.
Figure S21. UPLC chromatogram of purified cyclic lipopeptide **C_{10}-cTurg-2**.
Figure S22. UPLC chromatogram of purified cyclic lipopeptide **C_{12}-cTurg-2**.
Figure S23. UPLC chromatogram of purified cyclic lipopeptide **C_8-cTurg-6**.
Figure S24. UPLC chromatogram of purified cyclic lipopeptide **C_{10}-cTurg-6**.
Figure S25. UPLC chromatogram of purified cyclic lipopeptide **C_{12}-cTurg-6**.
Figure S26. Kinetic of the effect on viability as measured by relative luminescence in *B. subtilis* (pCGLS11) treated with different concentrations of **cTurg-2**, **cTurg-3**, **cTurg-4**, **cTurg-5**, **cTurg-6** and **cTurg-7**.

Figure S27. Kinetic of the effect on viability as measured by relative luminescence in *B. subtilis* (pCGLS11) treated with different concentrations of **C_8-cTurg-1**, **C_{10}-cTurg-1**, **C_{10}-cTurg-2**, **C_{12}-cTurg-2**, **C_8-cTurg-6**, **C_{10}-cTurg-6**, and **C_{12}-cTurg-6**.

Figure S28. Kinetic of the effect on membrane integrity as measured by relative luminescence in *B. subtilis* (pCSS962) treated with different concentrations **of cTurg-1**, **cTurg-2**, **cTurg-3**, **cTurg-4**, **cTurg-5**, **cTurg-6** and **cTurg-7**.

Figure S29. Kinetic of the effect on membrane integrity as measured by relative luminescence in *B. subtilis* (pCSS962) treated with different concentrations of **C_8-cTurg-1**, **C_{10}-cTurg-1**, **C_{10}-cTurg-2**, **C_{12}-cTurg-2**, **C_8-cTurg-6**, **C_{10}-cTurg-6**, and **C_{12}-cTurg-6**.

Figure S30. Kinetic of the effect on viability as measured by relative luminescence in *E. coli* (pCGLS-11) treated with 50 μg/mL of **cTurg-1-7** and 25 μg/mL of **chlorhexidine**.

Figure S31. Kinetic of the effect on viability as measured by relative luminescence in *E. coli* (pCGLS-11) treated with different concentrations **of C_8-cTurg-1**, **C_{10}-cTurg-1**, **C_{10}-cTurg-2**, **C_{12}-cTurg-2**, **C_8-cTurg-6**, **C_{10}-cTurg-6**, and **C_{12}-cTurg-6**.

Figure S32. Kinetic of the effect on membrane integrity as measured by relative luminescence in *E. coli* (pCSS962) treated with different concentrations of cyclic peptides **cTurg-1**, **cTurg-2**, **cTurg-3**, **cTurg-4**, **cTurg-5**, **cTurg-6** and **cTurg-7**.

Figure S33. Kinetic of the effect on membrane integrity as measured by relative luminescence in *E. coli* (pCSS962) treated with different concentrations of **C_8-cTurg-1**, **C_{10}-cTurg-1**, **C_{10}-cTurg-2**, **C_{12}-cTurg-2**, **C_8-cTurg-6**, **C_{10}-cTurg-6**, and **C_{12}-cTurg-6**.

Table S1. Theoretical and measured monoisotopic mass (Da), and theoretical and observed m/z ions during HRMS of the synthesised peptides.

Table S2. Purity of synthesized peptides (%) and retention time (min), determined by UPLC using a reversed phase column.

Table S3. Selectivity index (SI) calculated as the ration between haemolytic activity (EC_{50}, in μg/mL) and the geometric mean (GM) of the MIC values (in μg/mL) against bacteria and fungi, i.e., SI = EC_{50} / GM.

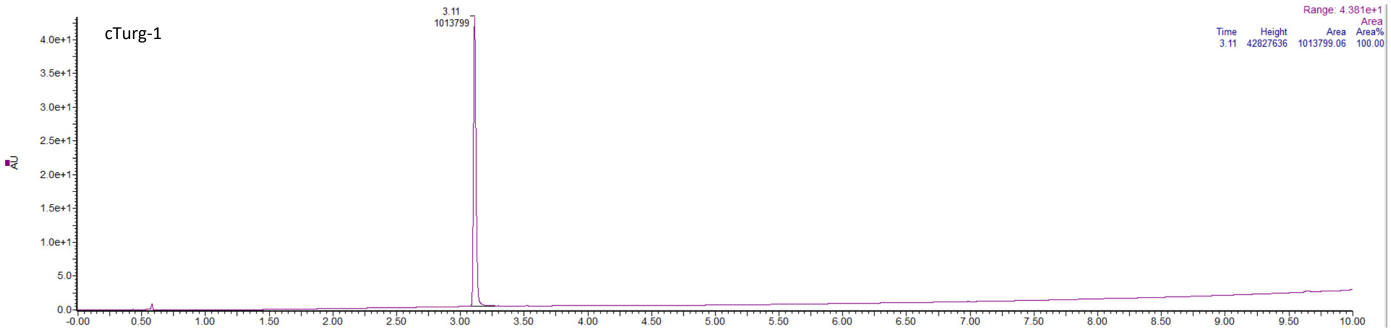

Figure S1. UPLC chromatogram of purified cyclic peptide **cTurg-1**. The peptide purity is 100 % based on the UPLC calculated area under the curves.

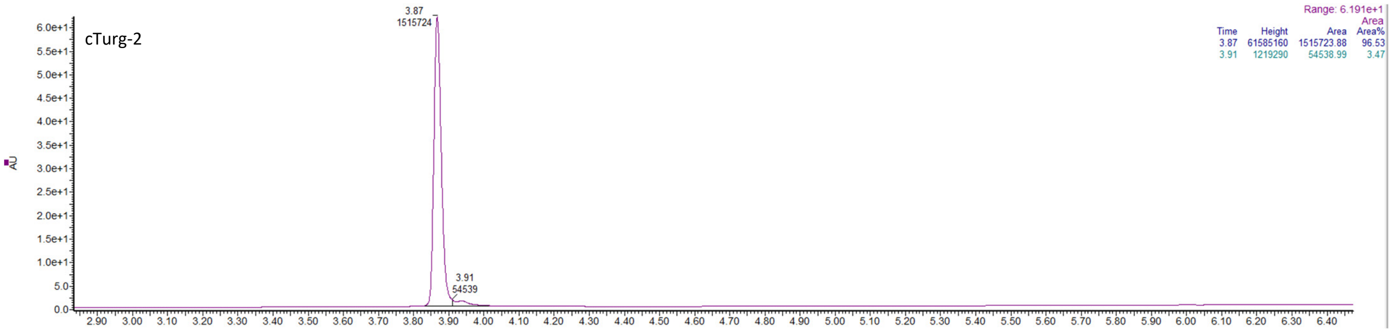

Figure S2. UPLC chromatogram of purified cyclic peptide **cTurg-2**. The peptide purity is 96.53 % based on the UPLC calculated area under the curves.

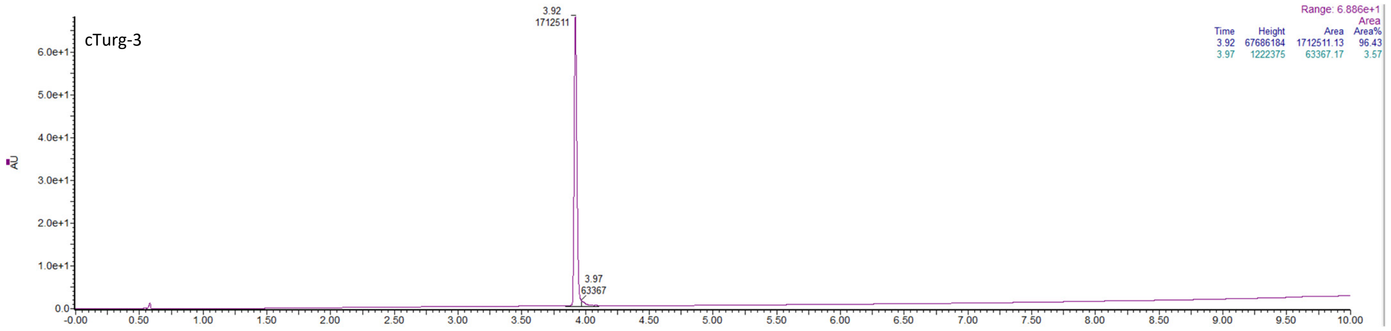

Figure S3. UPLC chromatogram of purified cyclic peptide **cTurg-3**. The peptide purity is 96.43 % based on the UPLC calculated area under the curves.

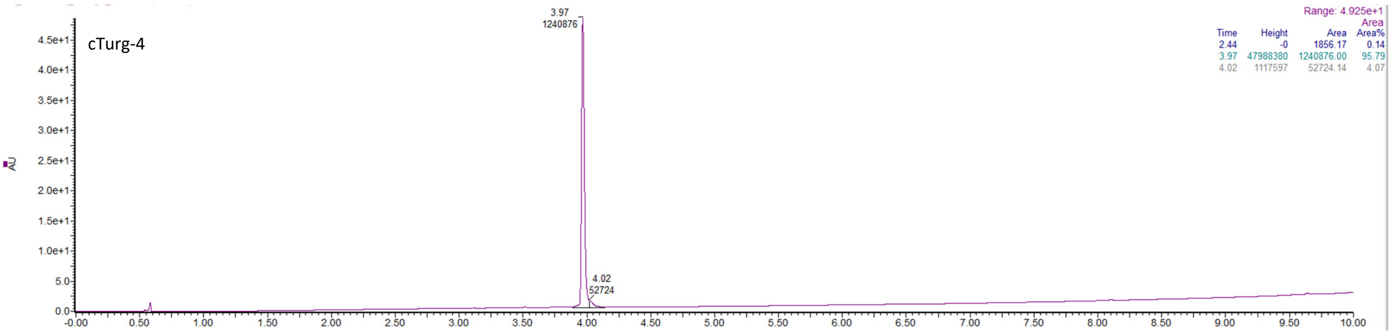

Figure S4. UPLC chromatogram of purified cyclic peptide **cTurg-4**. The peptide purity is 95.79 % based on the UPLC calculated area under the curves.

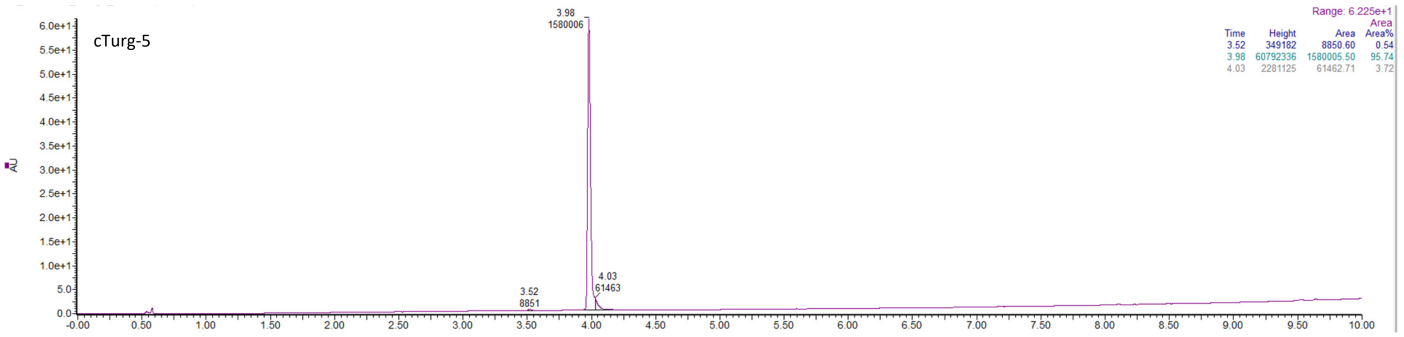

Figure S5. UPLC chromatogram of purified cyclic peptide **cTurg-5**. The peptide purity is 95.74 % based on the UPLC calculated area under the curves.

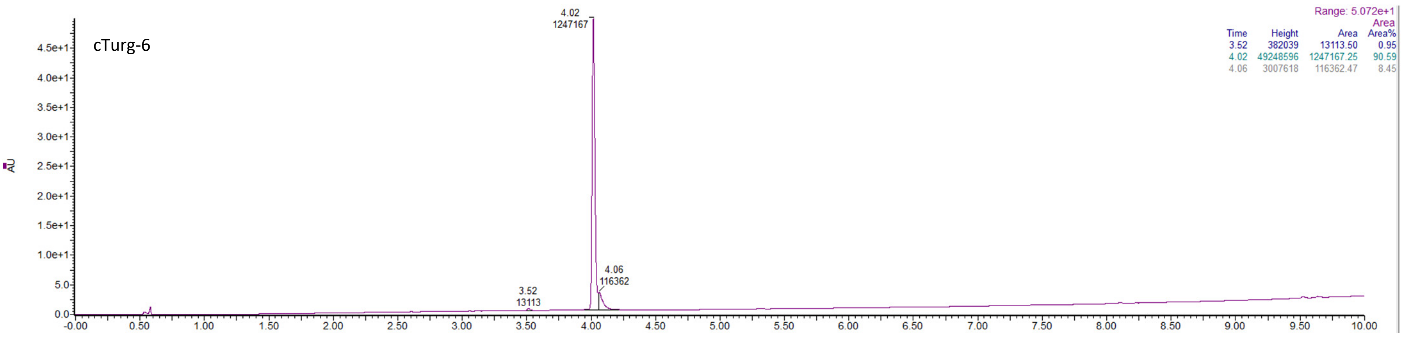

Figure S6. UPLC chromatogram of purified cyclic peptide **cTurg-6**. The peptide purity is 90.59 % based on the UPLC calculated area under the curves.

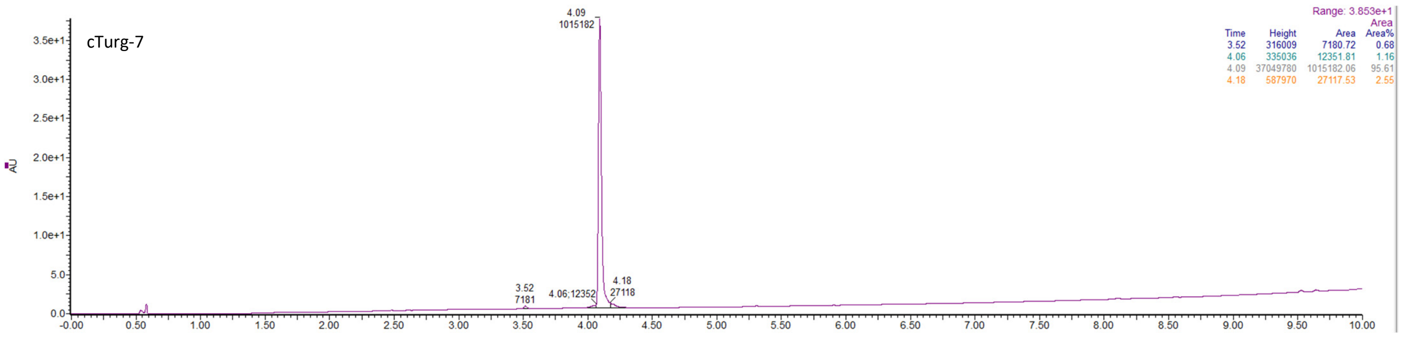

Figure S7. UPLC chromatogram of purified cyclic peptide **cTurg-7**. The peptide purity is 95.61 % based on the UPLC calculated area under the curves.

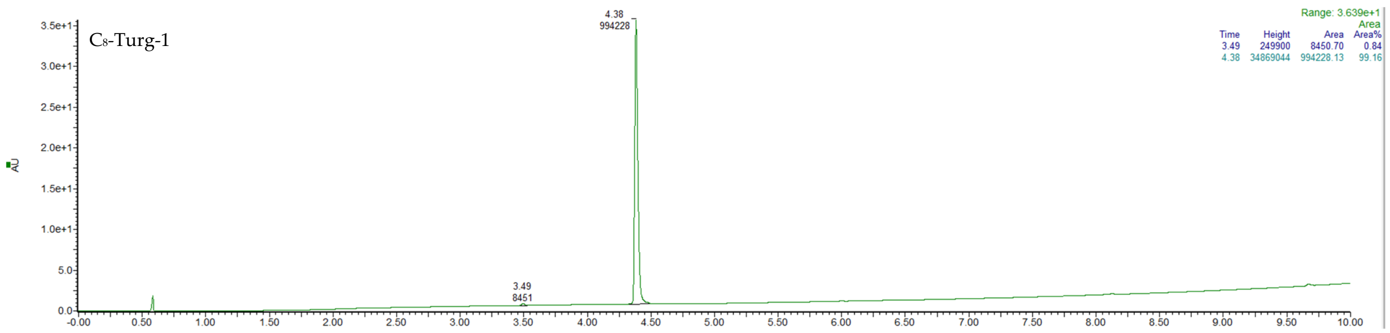

Figure S8. UPLC chromatogram of purified linear lipopeptide **C_8-Turg-1**. The peptide purity is 99.16 % based on the UPLC calculated area under the curves.

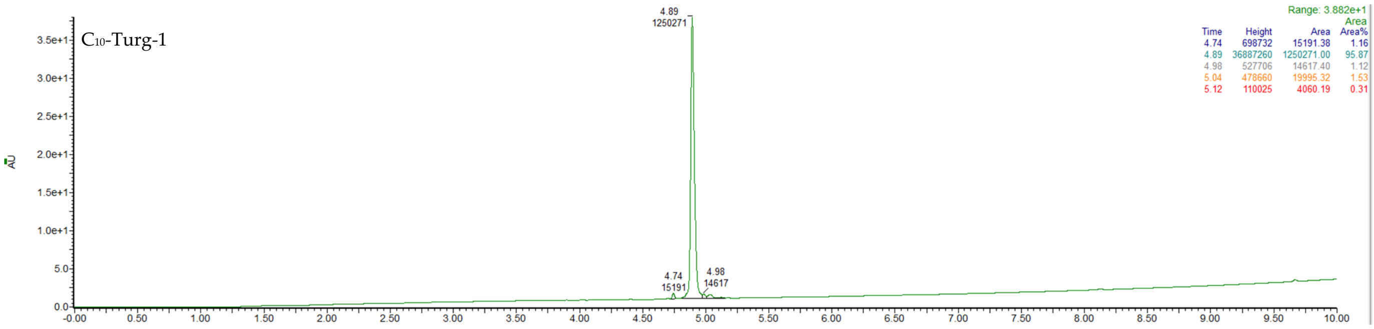

Figure S9. UPLC chromatogram of purified linear lipopeptide **C10-Turg-1**. The peptide purity is 95.87 % based on the UPLC calculated area under the curves.

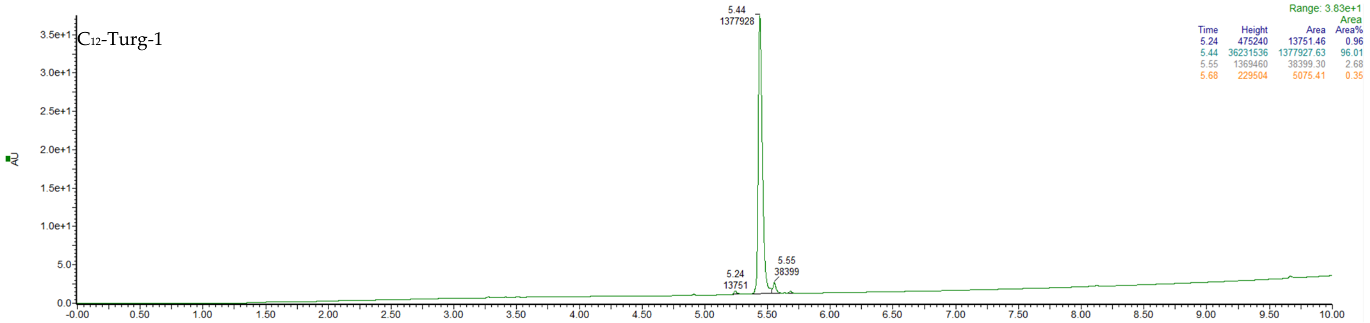

Figure S10. UPLC chromatogram of purified linear lipopeptide **C12-Turg-1**. The peptide purity is 96.01 % based on the UPLC calculated area under the curves.

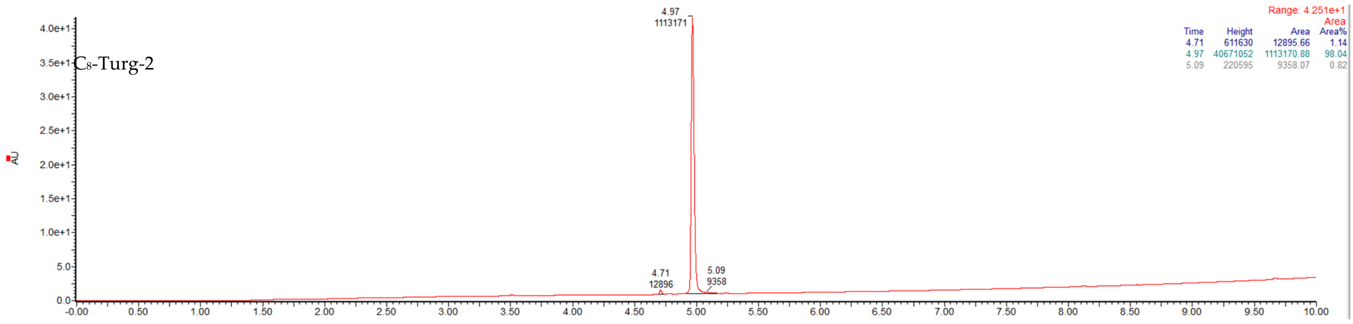

Figure S11. UPLC chromatogram of purified linear lipopeptide **C8-Turg-2**. The peptide purity is 98.04 % based on the UPLC calculated area under the curves.

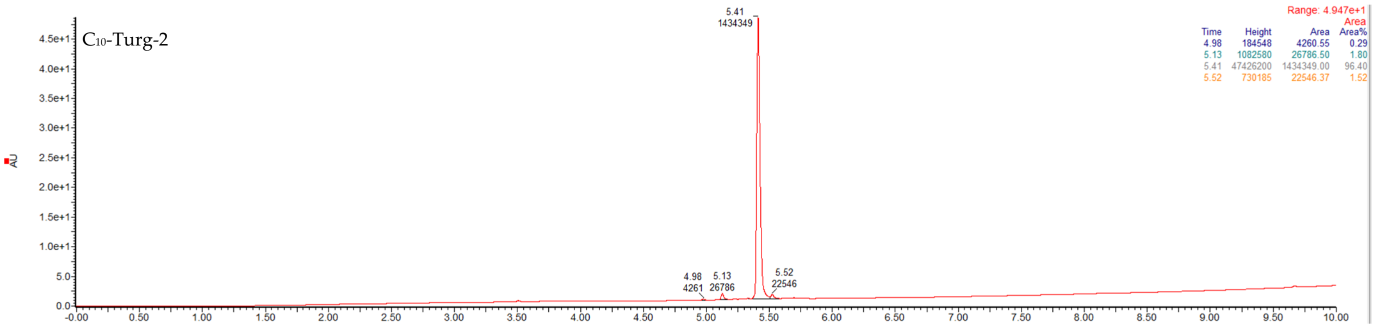

Figure S12. UPLC chromatogram of purified linear lipopeptide **C10-Turg-2**. The peptide purity is 96.40 % based on the UPLC calculated area under the curves.

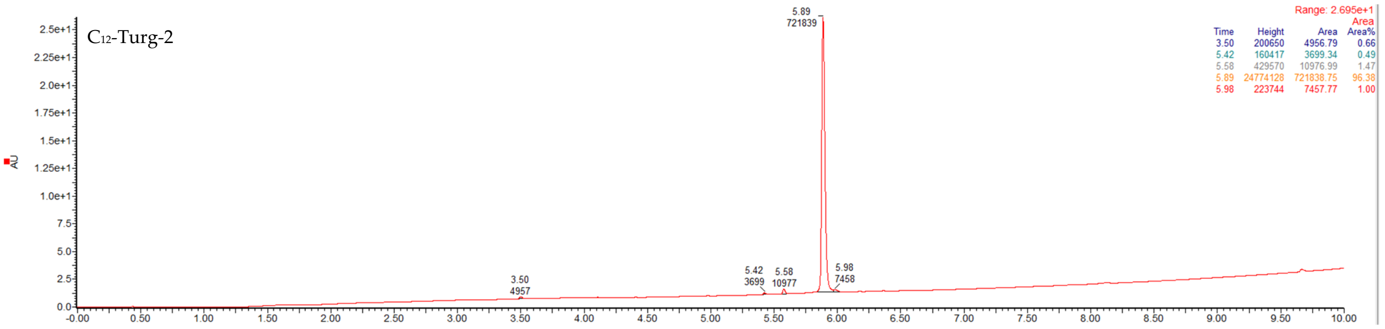

Figure S13. UPLC chromatogram of purified linear lipopeptide **C12-Turg-2**. The peptide purity is 96.38 % based on the UPLC calculated area under the curves.

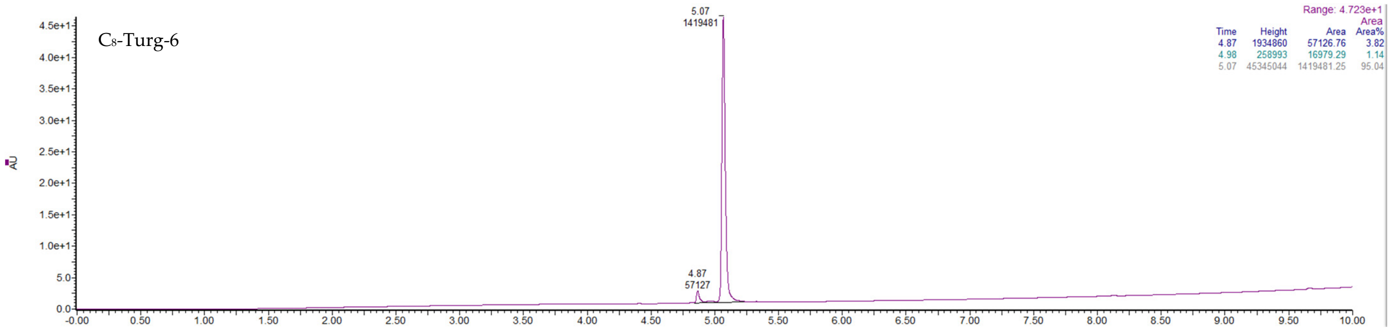

Figure S14. UPLC chromatogram of purified linear lipopeptide **C8-Turg-6**. The peptide purity is 95.04 % based on the UPLC calculated area under the curves.

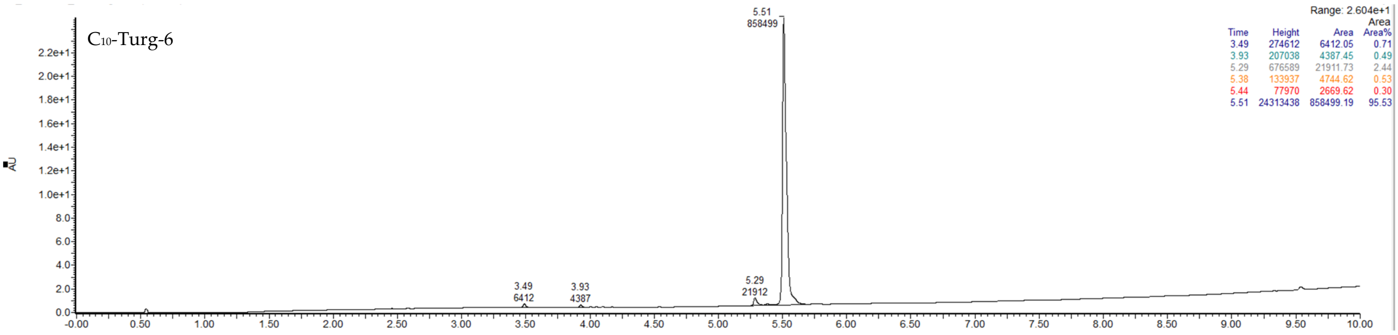

Figure S15. UPLC chromatogram of purified linear lipopeptide **C10-Turg-6**. The peptide purity is 95.53 % based on the UPLC calculated area under the curves.

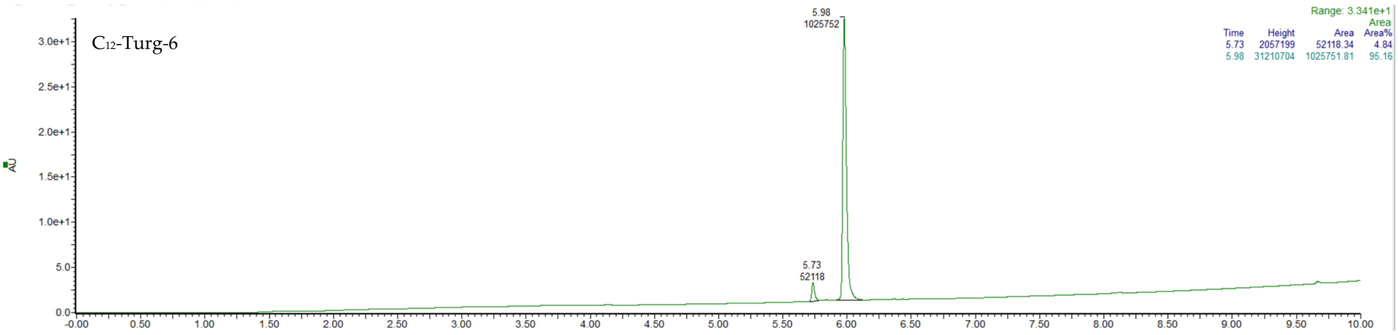

Figure S16. UPLC chromatogram of purified linear lipopeptide **C12-Turg-6**. The peptide purity is 95.16 % based on the UPLC calculated area under the curves.

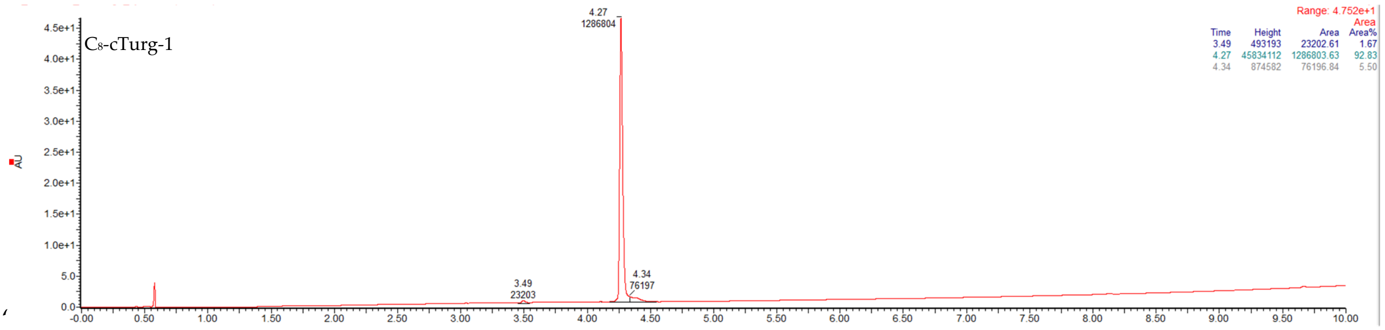

Figure S17. UPLC chromatogram of purified cyclic lipopeptide **C8-cTurg-1**. The peptide purity is 92.83 % based on the UPLC calculated area under the curves.

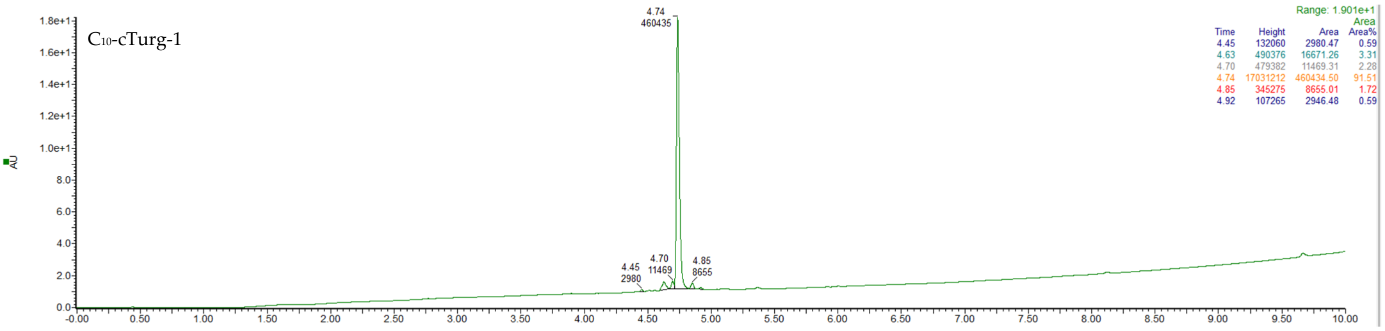

Figure S18. UPLC chromatogram of purified cyclic lipopeptide **C10-cTurg-1**. The peptide purity is 91.51 % based on the UPLC calculated area under the curves.

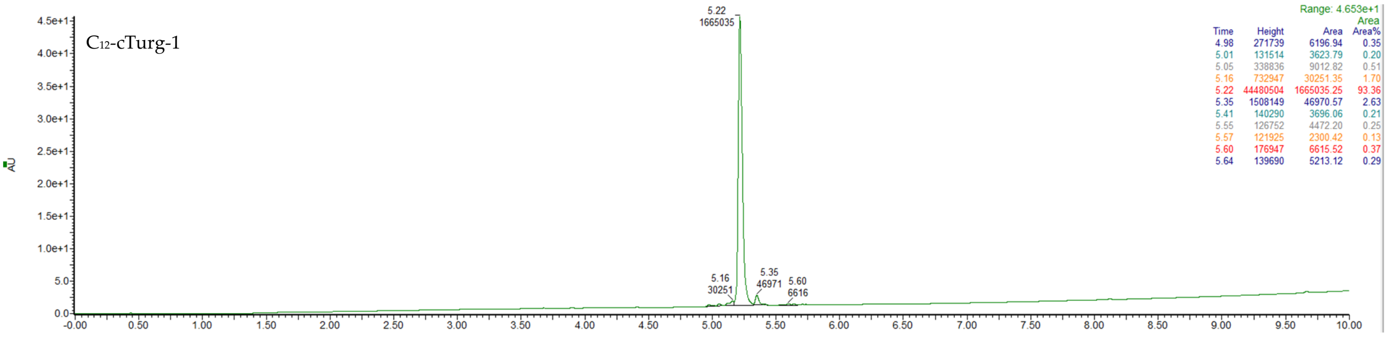

Figure S19. UPLC chromatogram of purified cyclic lipopeptide **C12-cTurg-1**. The peptide purity is 93.36 % based on the UPLC calculated area under the curves.

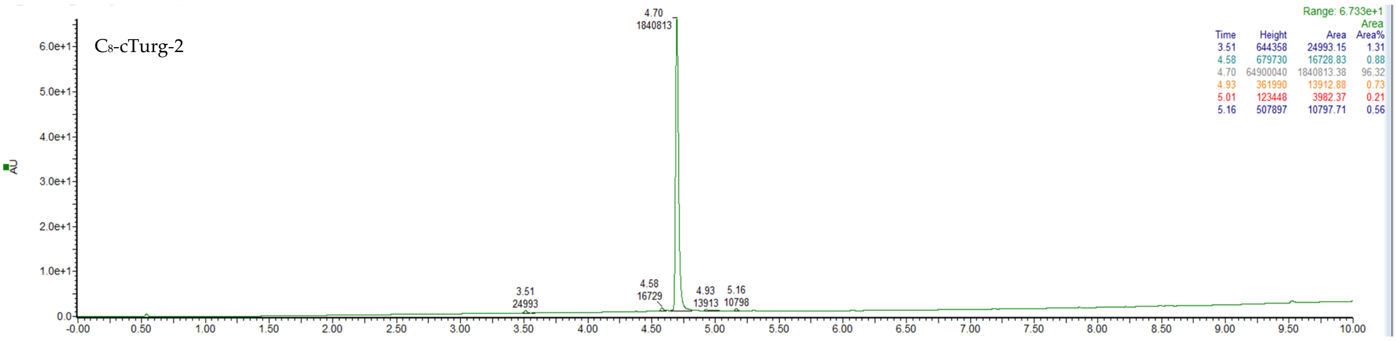

Figure S20. UPLC chromatogram of purified cyclic lipopeptide **C8-cTurg-2**. The peptide purity is 96.32 % based on the UPLC calculated area under the curves.

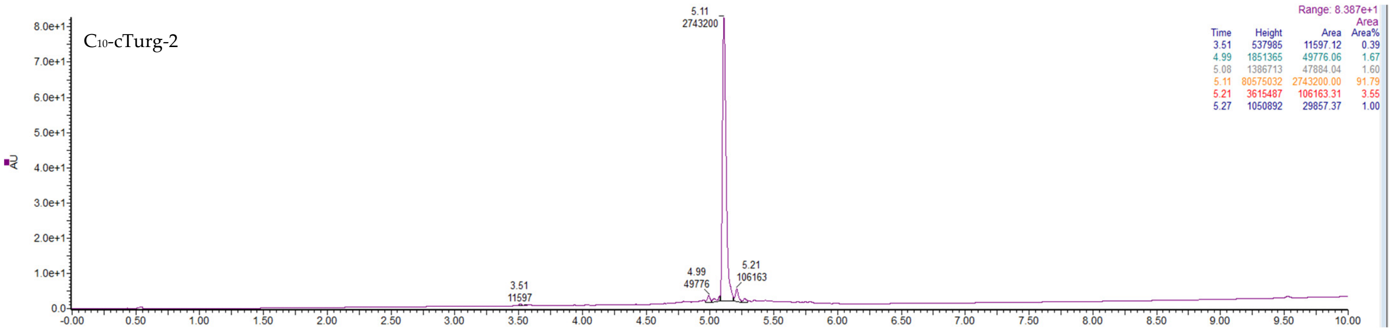

Figure S21. UPLC chromatogram of purified cyclic lipopeptide **C10-cTurg-2**. The peptide purity is 91.79 % based on the UPLC calculated area under the curves.

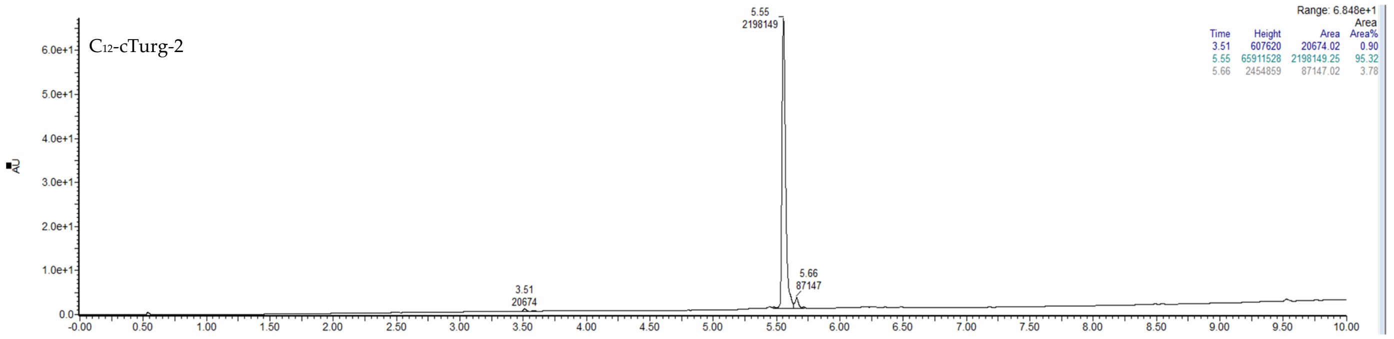

Figure S22. UPLC chromatogram of purified cyclic lipopeptide **C12-cTurg-2**. The peptide purity is 95.32 % based on the UPLC calculated area under the curves.

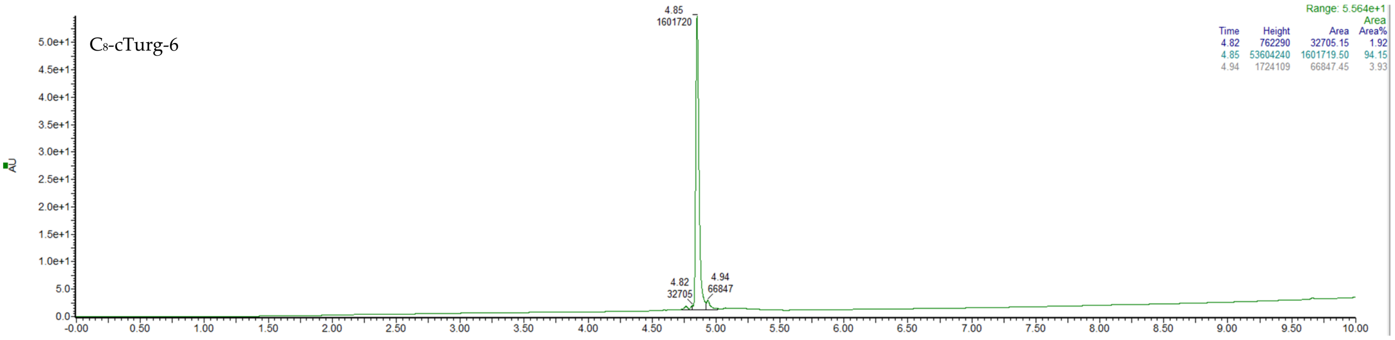

Figure S23. UPLC chromatogram of purified cyclic lipopeptide **C8-cTurg-6**. The peptide purity is 94.15 % based on the UPLC calculated area under the curves.

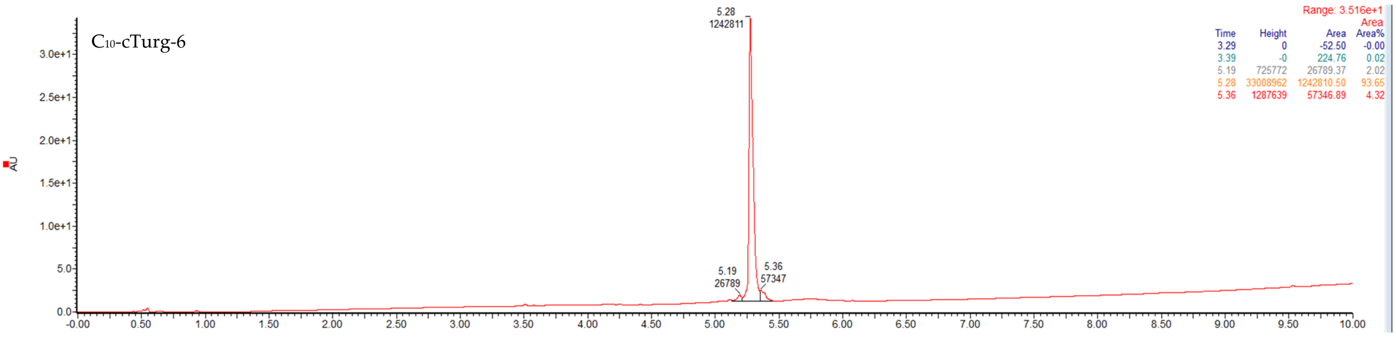

Figure S24. UPLC chromatogram of purified cyclic lipopeptide **C10-cTurg-6**. The peptide purity is 93.65 % based on the UPLC calculated area under the curves.

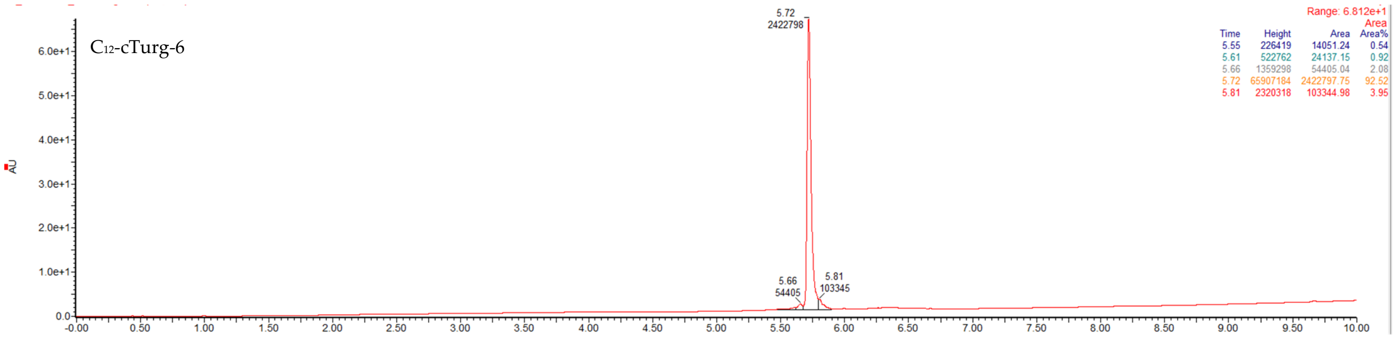

Figure S25. UPLC chromatogram of purified cyclic lipopeptide **C12-cTurg-6**. The peptide purity is 92.52 % based on the UPLC calculated area under the curves.

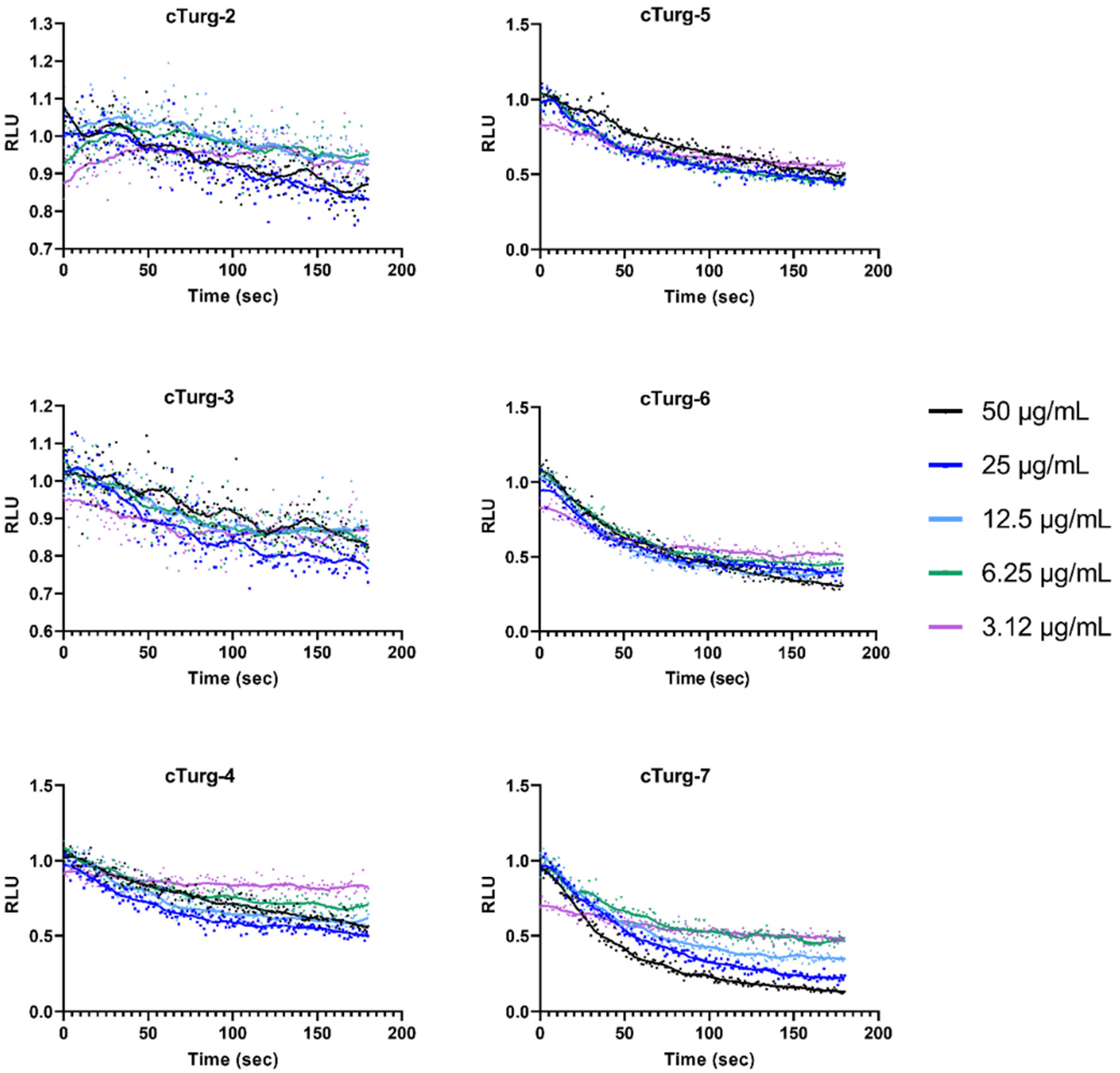

Figure S26. Kinetic of the effect on viability as measured by relative luminescence in *B. subtilis* (pCGLS11) treated with different concentrations of **cTurg-2**, **cTurg-3**, **cTurg-4**, **cTurg-5**, **cTurg-6** and **cTurg-7**.

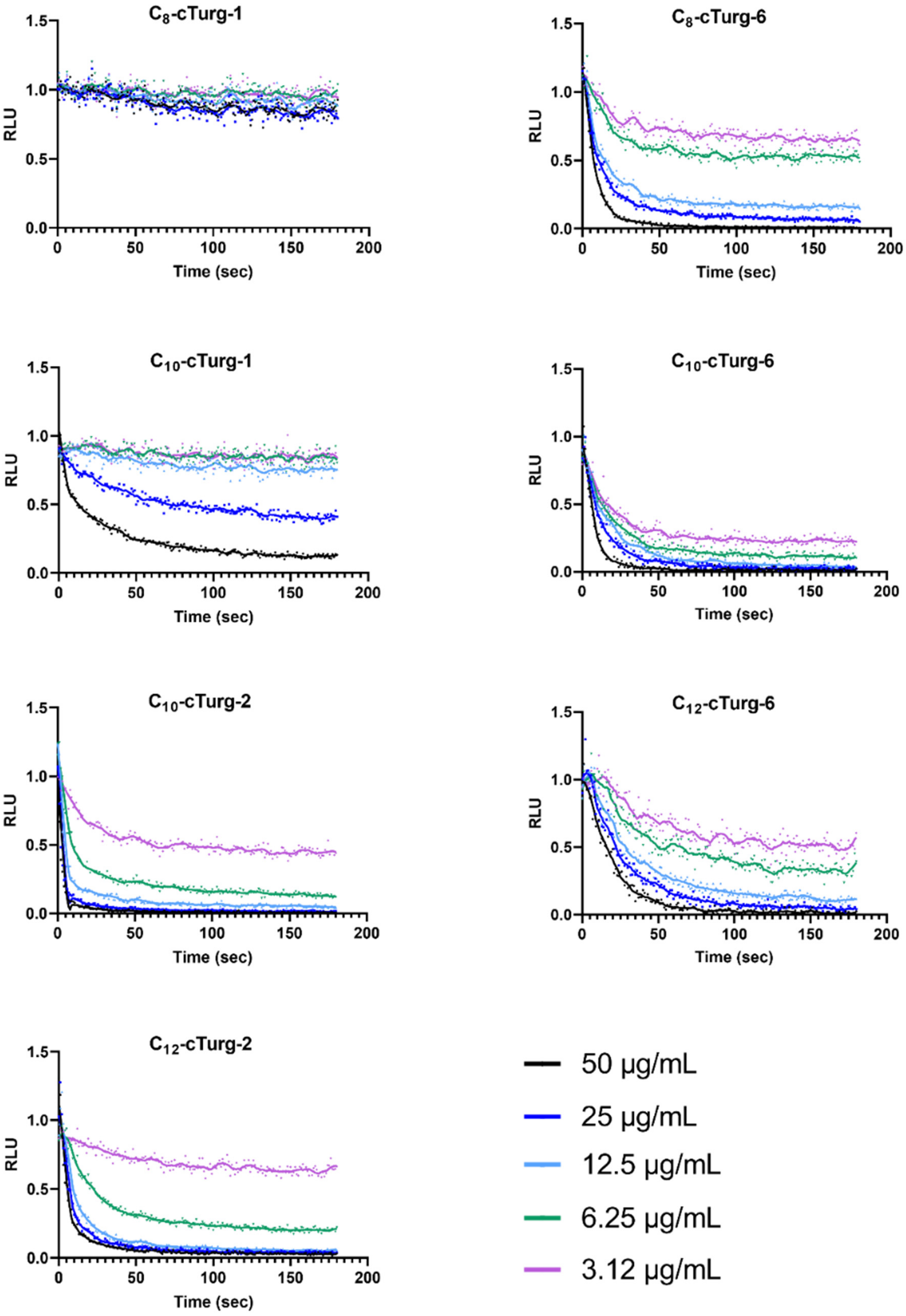

Figure S27. Kinetic of the effect on viability as measured by relative luminescence in *B. subtilis* (pCGLS11) treated with different concentrations of **C_8-cTurg-1**, **C_{10}-cTurg-1**, **C_{10}-cTurg-2**, **C_{12}-cTurg-2**, **C_8-cTurg-6**, **C_{10}-cTurg-6**, and **C_{12}-cTurg-6**.

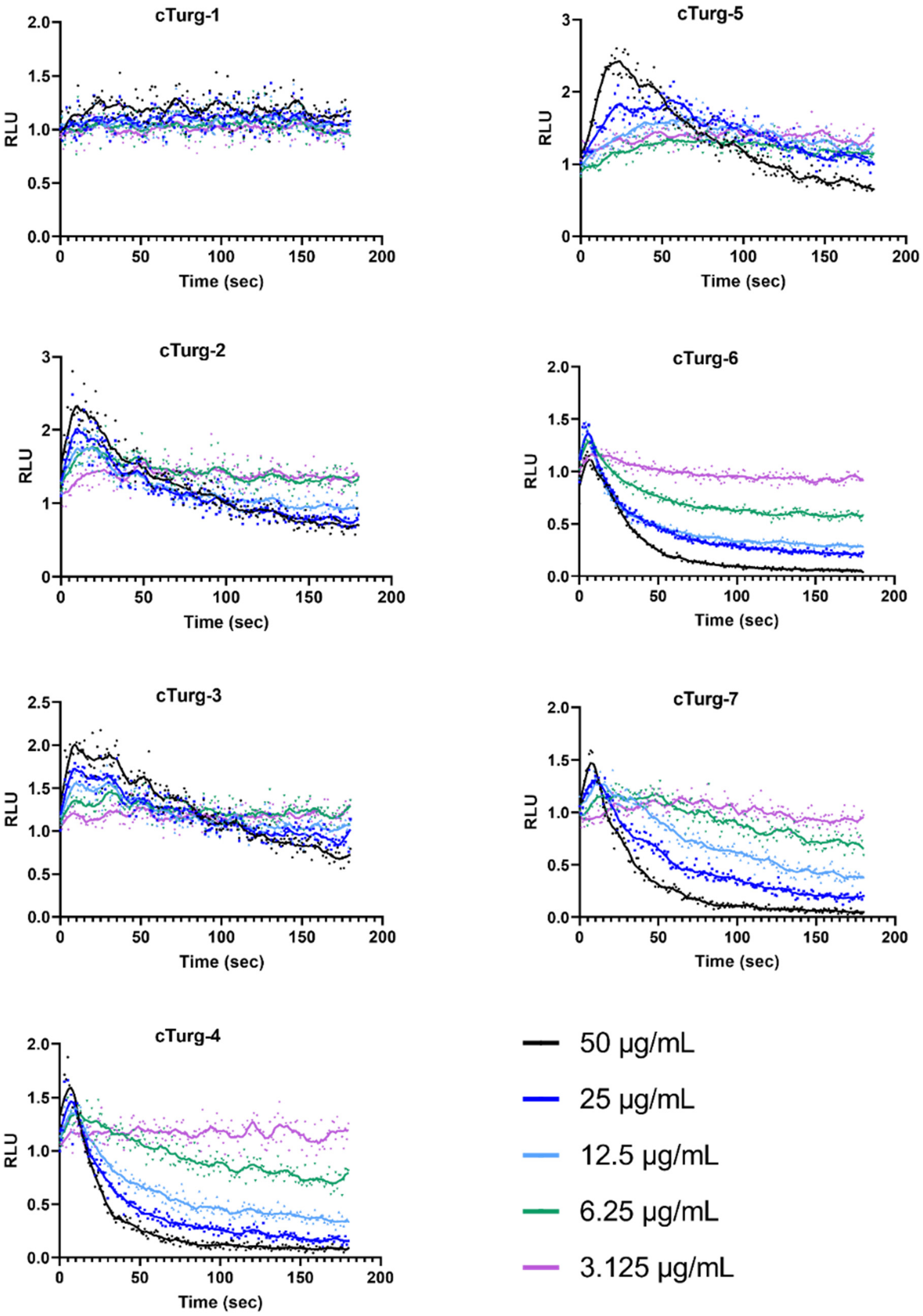

Figure S28. Kinetic of the effect on membrane integrity as measured by relative luminescence in *B. subtilis* (pCSS962) treated with different concentrations of **cTurg-1**, **cTurg-2**, **cTurg-3**, **cTurg-4**, **cTurg-5**, **cTurg-6** and **cTurg-7**.

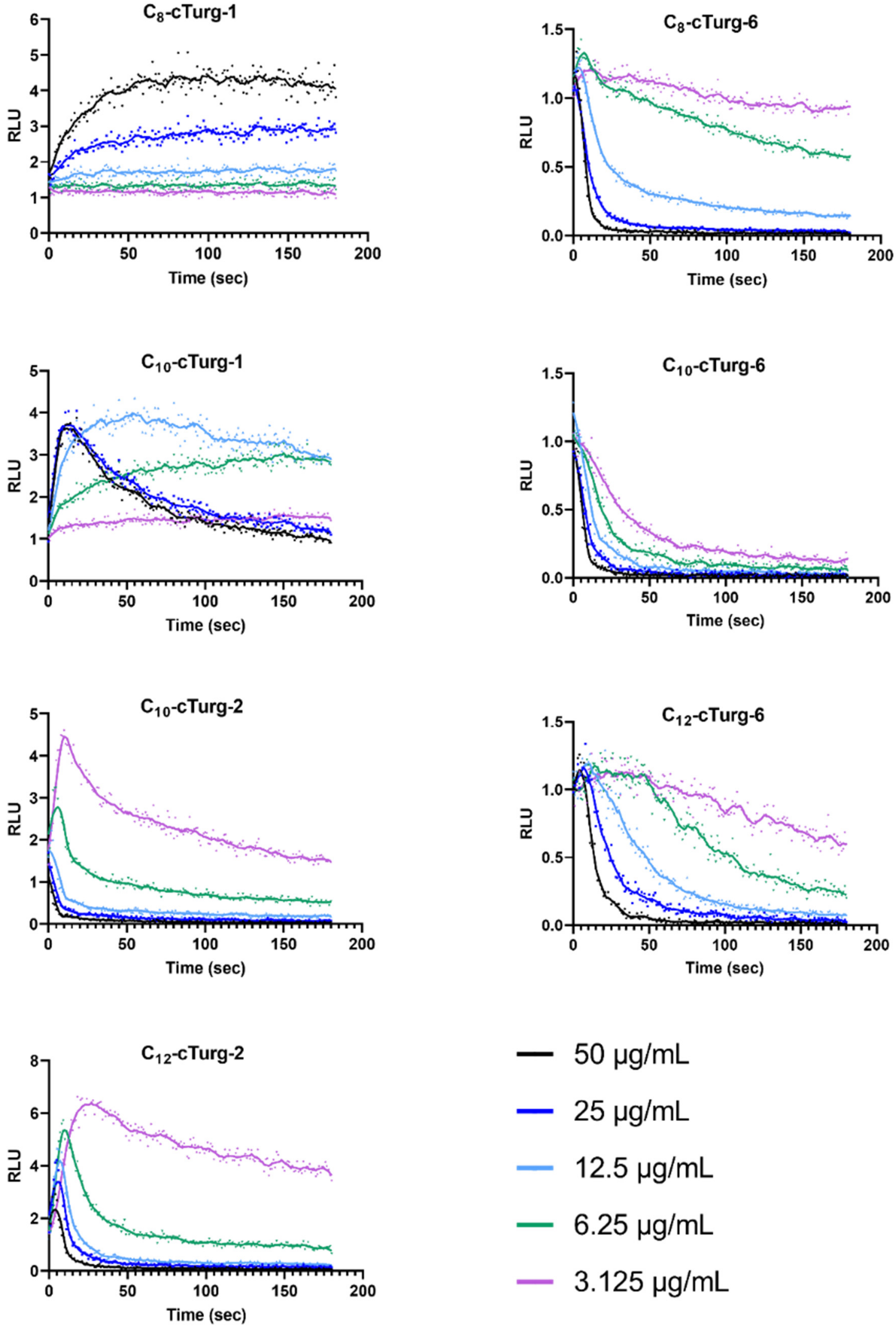

Figure S29. Kinetic of the effect on membrane integrity as measured by relative luminescence in *B. subtilis* (pCSS962) treated with different concentrations of **C_8-cTurg-1**, **C_{10}-cTurg-1**, **C_{10}-cTurg-2**, **C_{12}-cTurg-2**, **C_8-cTurg-6**, **C_{10}-cTurg-6**, and **C_{12}-cTurg-6**.

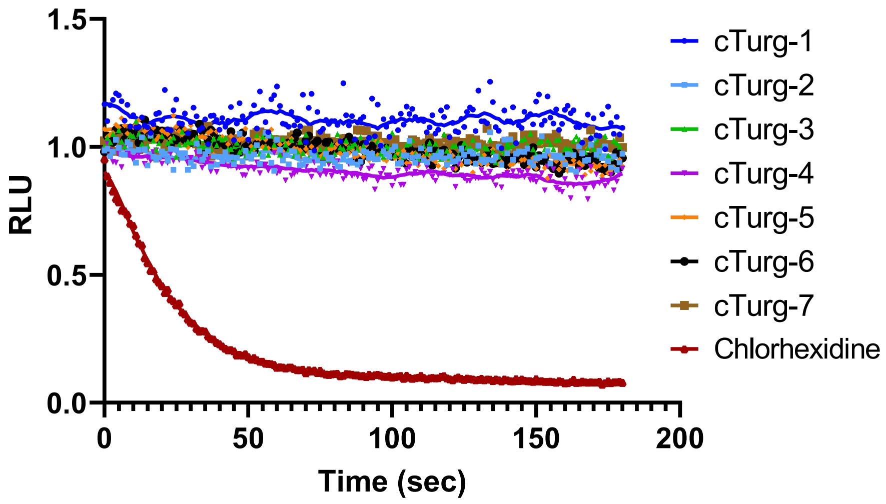

Figure S30. Kinetic of the effect on viability as measured by relative luminescence in *E. coli* (pCGLS-11) treated with 50 μg/mL of **cTurg-1-7** or 25 μg/mL of **chlorhexidine**.

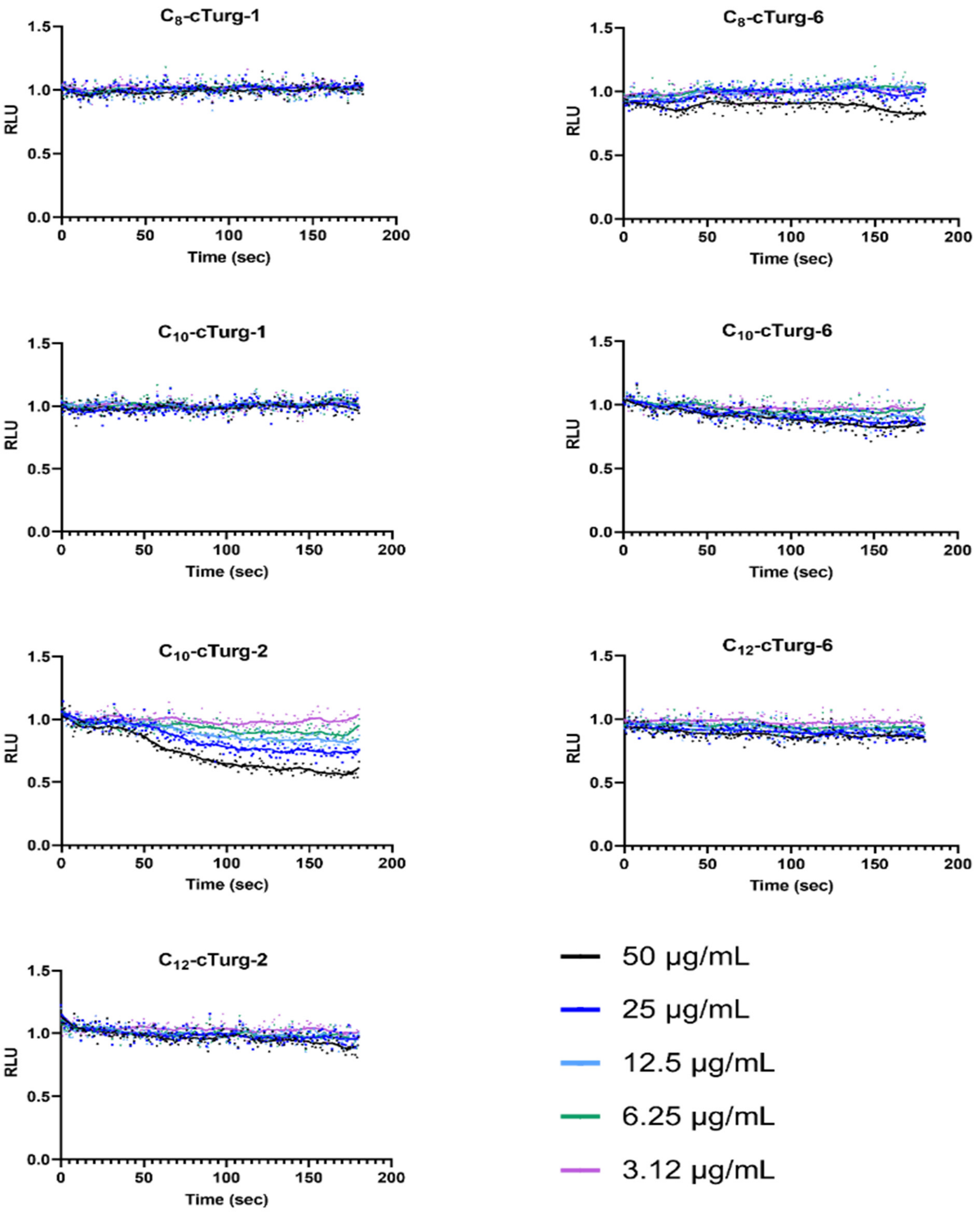

Figure S31. Kinetic of the effect on viability as measured by relative luminescence in *E. coli* (pCGLS-11) treated with different concentrations of **C_8-cTurg-1**, **C_{10}-cTurg-1**, **C_{10}-cTurg-2**, **C_{12}-cTurg-2**, **C_8-cTurg-6**, **C_{10}-cTurg-6**, and **C_{12}-cTurg-6**.

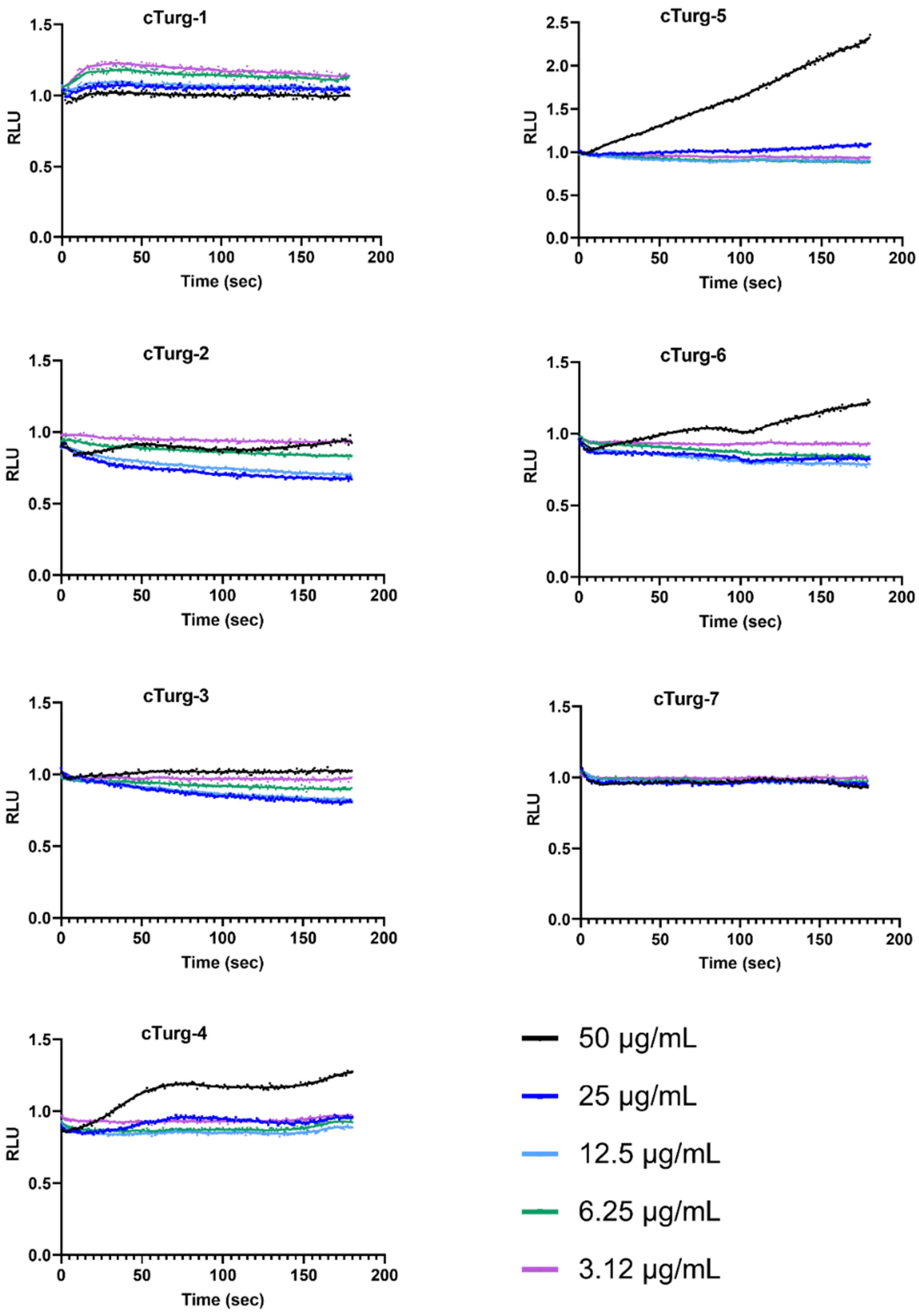

Figure S32. Kinetic of the effect on membrane integrity as measured by relative luminescence in *E. coli* (pCSS962) treated with different concentrations of cyclic peptides **cTurg-1**, **cTurg-2**, **cTurg-3**, **cTurg-4**, **cTurg-5**, **cTurg-6** and **cTurg-7**.

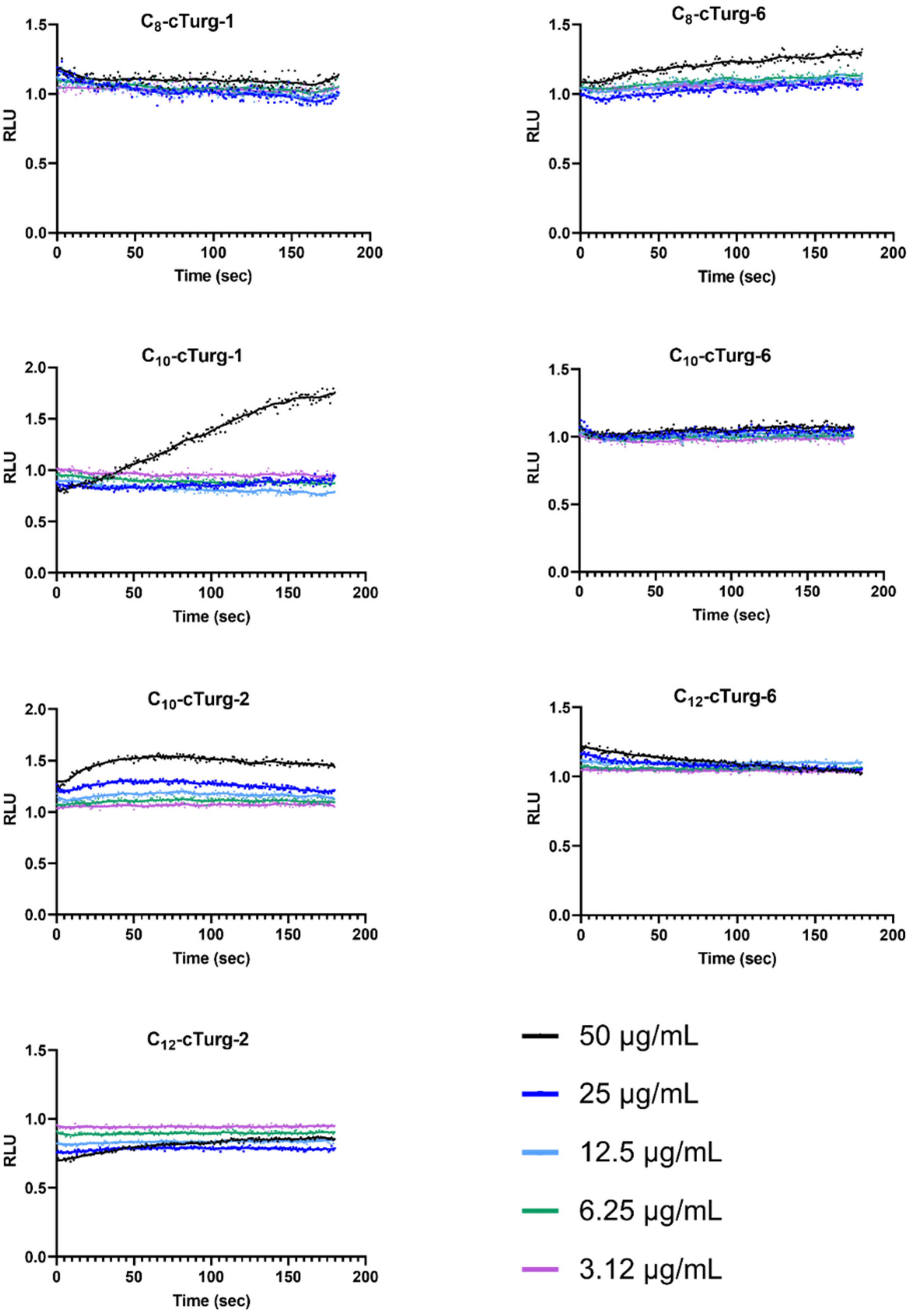

Figure S33. Kinetic of the effect on membrane integrity as measured by relative luminescence in *E. coli* (pCSS962) treated with different concentrations of **C8-cTurg-1**, **C10-cTurg-1**, **C10-cTurg-2**, **C12-cTurg-2**, **C8-cTurg-6**, **C10-cTurg-6**, **and C12-cTurg-6**.

Table S1. Theoretical and measured monoisotopic mass (Da), and theoretical and observed m/z ions during HRMS of the synthesised peptides.

	**Monoisotopic mass (Da)**		**$[M+2H]^{2+}$**		**$[M+3H]^{3+}$**		**$[M+4H]^{4+}$**	
**Peptide**	Theoretical	Measured	Theoretical	Measured	Theoretical	Measured	Theoretical	Measured
**cTurg-1**	1300.6897	1300.6915	651.3521	651.3525	434.5705	434.5713	326.1797	326.1803
**cTurg-2**	1518.7741	1518.7746	760.3943	760.3944	507.2653	507.2656	380.7008	380.7009
**cTurg-3**	1518.7741	1518.7746	760.3943	760.3944	507.2653	507.2656	380.7008	380.7009
**cTurg-4**	1558.8054	1558.8061	780.4100	780.4102	520.6091	520.6094	390.7086	390.7088
**cTurg-5**	1630.7986	1630.7993	816.4066	816.4068	544.6068	544.6071	408.7069	408.7071
**cTurg-6**	1630.7987	1630.7999	816.4066	816.4068	544.6068	544.6073	408.7069	408.7074
**cTurg-7**	1670.8299	1670.8308	836.4222	836.4222	557.9506	557.9509	418.7148	418.7152
**C_8-Turg-1**	1428.8098	1428.8104	715.4122	715.4123	477.2772	477.2775	358.2097	358.2099
**C_{10}-Turg-1**	1456.8411	1456.8415	729.4278	729.4279	486.6210	486.6211	365.2176	365.2177
**C_{12}-Turg-1**	1484.8724	1484.8732	743.4435	743.4437	495.9647	495.9651	372.2254	372.2256
**C_8-Turg-2**	1646.8942	1646.8950	824.4544	824.4544	549.9720	549.9724	412.7308	412.7311
**C_{10}-Turg-2**	1674.9255	1674.9254	838.4700	838.4695	559.3158	559.3158	419.7387	419.7388
**C_{12}-Turg-2**	1702.9568	1702.9566	852.4857	852.4851	568.6595	568.6597	426.7465	426.7465
**C_8-Turg-6**	1758.9188	1758.9195	880.4667	880.4666	587.3135	587.3139	440.7370	440.7373
**C_{10}-Turg-6**	1786.9501	1786.9515	894.4823	894.4827	596.6573	596.6579	447.7448	447.7452
**C_{12}-Turg-6**	1814.9814	1814.9829	908.4980	908.4981	606.0011	606.0019	454.7526	454.7531
**C_8-cTurg-1**	1426.7941	1426.7948	714.4043	714.4045	476.6053	476.6056	357.7058	357.7060
**C_{10}-cTurg-1**	1454.8254	1454.8257	728.4200	728.4200	485.9491	485.9493	364.7136	364.7137
**C_{12}-cTurg-1**	1482.8567	1482.8573	742.4356	742.4358	495.2928	495.2931	371.7215	371.7216
**C_8-cTurg-2**	1644.8785	1644.8805	823.4465	823.4470	549.3001	549.3013	412.2269	412.2273
**C_{10}-cTurg-2**	1672.9098	1672.9107	837.4622	837.4621	558.6439	558.6446	419.2347	419.2349
**C_{12}-cTurg-2**	1700.9411	1700.9422	851.4778	851.4775	567.9876	567.9888	426.2426	426.2427
**C_8-cTurg-6**	1756.9031	1756.9036	879.4588	879.4586	586.6416	586.6420	440.2331	440.2333
**C_{10}-cTurg-6**	1784.9344	1784.9351	893.4745	893.4743	595.9854	595.9860	447.2409	447.2411
**C_{12}-cTurg-6**	1812.9657	1812.9685	907.4901	907.4905	605.3292	605.3303	454.2487	454.2498

Table S2. Purity of synthesized peptides (%) and retention time (min), determined by UPLC using a reversed phase column.

Peptide	Sequence	Purity [%]	Retention time [min]
**cTurg-1**	cyclic	100.00	3.11
**cTurg-2**	cyclic	96.53	3.87
**cTurg-3**	cyclic	96.43	3.92
**cTurg-4**	cyclic	95.79	3.97
**cTurg-5**	cyclic	95.74	3.98
**cTurg-6**	cyclic	90.59	4.02
**cTurg-7**	cyclic	95.61	4.09
**C_8-Turg-1**	linear	99.16	4.38
**C_{10}-Turg-1**	linear	95.87	4.89
**C_{12}-Turg-1**	linear	96.01	5.44
**C_8-Turg-2**	linear	98.04	4.97
**C_{10}-Turg-2**	linear	96.40	5.41
**C_{12}-Turg-2**	linear	96.38	5.89
**C_8-Turg-6**	linear	95.04	5.07
**C_{10}-Turg-6**	linear	95.53	5.51
**C_{12}-Turg-6**	linear	95.16	5.98
**C_8-cTurg-1**	cyclic	92.83	4.27
**C_{10}-cTurg-1**	cyclic	91.51	4.74
**C_{12}-cTurg-1**	cyclic	93.36	5.22
**C_8-cTurg-2**	cyclic	96.32	4.70
**C_{10}-cTurg-2**	cyclic	91.79	5.11
**C_{12}-cTurg-2**	cyclic	95.32	5.55
**C_8-cTurg-6**	cyclic	94.15	4.85
**C_{10}-cTurg-6**	cyclic	93.65	5.28
**C_{12}-cTurg-6**	cyclic	92.52	5.72

Table S3. Selectivity index (SI) calculated as the ration between haemolytic activity (EC_{50}, in µg/mL) and the geometric mean (GM) of the MIC values (in µg/mL) against bacteria and fungi, i.e., SI = EC_{50} / GM. MIC >256 were set to 256 for bacteria, MIC >128 were set to 128 for fungi, and the values for the highest tested concentration was used for haemolytic activity when calculating the SI.

			GM of MIC				Selectivity index (SI)			
		**Peptide**	**G+**	**G-**	**Tot. bact.**[1]	**Fungi**	**G+**	**G-**	**Tot. bact.**	**Fungi**
**Cyclic peptides**		**cTurg-1**	128	256	161	64	nd[2]	nd	nd	nd
	**W**	**cTurg-2**	11	64	20	32	92	16	52	33
	**W**	**cTurg-3**	10	64	18	32	89	13	47	27
	**W**	**cTurg-4**	16	128	32	40	67	8	33	26
	**R / W**	**cTurg-5**	8	11	9	32	138	97	123	34
	**R / W**	**cTurg-6**	7	11	8	32	164	97	138	34
	**R / W**	**cTurg-7**	7	23	10	32	29	9	20	6
**Linear lipopeptides**		**C_8-Turg-1**	19	64	29	40	50	15	33	23
		**C_{10}-Turg-1**	7	23	10	25	142	42	95	38
		**C_{12}-Turg-1**	5	11	6	25	204	86	153	38
	**W**	**C_8-Turg-2**	7	16	9	40	29	12	22	5
	**W**	**C_{10}-Turg-2**	7	11	8	40	10	6	8	2
	**W**	**C_{12}-Turg-2**	11	23	14	40	5	2	4	1
	**R / W**	**C_8-Turg-6**	7	16	9	102	8	3	6	1
	**R / W**	**C_{10}-Turg-6**	16	45	23	102	1	0	1	0
	**R / W**	**C_{12}-Turg-6**	19	91	32	91	2	0	1	0
**Cyclic lipopeptides**		**C_8-cTurg-1**	16	64	25	32	59	15	37	29
		**C_{10}-cTurg-1**	4	16	6	20	239	60	151	47
		**C_{12}-cTurg-1**	3	8	4	20	77	27	55	11
	**W**	**C_8-cTurg-2**	3	6	4	13	155	78	123	35
	**W**	**C_{10}-cTurg-2**	3	8	5	32	32	13	24	3
	**W**	**C_{12}-cTurg-2**	7	16	9	40	5	2	4	1
	**R / W**	**C_8-cTurg-6**	5	11	6	51	6	3	5	1
	**R / W**	**C_{10}-cTurg-6**	6	16	8	51	3	1	2	0
	**R / W**	**C_{12}-cTurg-6**	10	32	14	81	1	0	1	0

[1] GM of all bacterial test strains, [2] nd: not determined.
